# Local Homing Navigation Based on the Moment Model for Landmark Distribution and Features

**DOI:** 10.3390/s17112658

**Published:** 2017-11-17

**Authors:** Changmin Lee, DaeEun Kim

**Affiliations:** School of Electrical and Electronic Engineering, Yonsei University, Seoul 03722, Korea; lcmin@yonsei.ac.kr

**Keywords:** local visual navigation, moment model, landmark vector, range sensor, homing navigation

## Abstract

For local homing navigation, an agent is supposed to return home based on the surrounding environmental information. According to the snapshot model, the home snapshot and the current view are compared to determine the homing direction. In this paper, we propose a novel homing navigation method using the moment model. The suggested moment model also follows the snapshot theory to compare the home snapshot and the current view, but the moment model defines a moment of landmark inertia as the sum of the product of the feature of the landmark particle with the square of its distance. The method thus uses range values of landmarks in the surrounding view and the visual features. The center of the moment can be estimated as the reference point, which is the unique convergence point in the moment potential from any view. The homing vector can easily be extracted from the centers of the moment measured at the current position and the home location. The method effectively guides homing direction in real environments, as well as in the simulation environment. In this paper, we take a holistic approach to use all pixels in the panoramic image as landmarks and use the RGB color intensity for the visual features in the moment model in which a set of three moment functions is encoded to determine the homing vector. We also tested visual homing or the moment model with only visual features, but the suggested moment model with both the visual feature and the landmark distance shows superior performance. We demonstrate homing performance with various methods classified by the status of the feature, the distance and the coordinate alignment.

## 1. Introduction

Navigation is a process of monitoring and controlling the movement of an agent from one place to another. Many navigation systems have their goal positions that the agent is supposed to reach. Recent navigation studies show diverse forms of moving vehicles from simple wheeled robots [1] to a wide range of underwater vehicles [2], unmanned aerial vehicles [3] and spacecraft [4] with various sensors [5,6]. Furthermore, there have been techniques using various sensors like vision [7], inertia [8] and RFID [9], which represent a great deal of engineering achievement.

Different from the above engineering approaches, various animals demonstrate remarkable homing capacity, and their navigation system robustly works in real environments. Local visual homing based on a snapshot model [10] is inspired by insect navigation. An agent is supposed to return to the nest using visual cues or landmarks. The snapshot model uses only a pair of snapshot images at the nest and at the current position. The difference of landmark positions in the two images can be used to derive information about the relative location difference or homing direction. The angular difference in landmark position can greatly contribute to decisions about homing. Honeybees can find the homing direction by reducing the differences of the angular distribution of visual landmarks observed in a pair of snapshots [10].

Desert ants (Cataglyphis fortis) are known to use odometry and visual information for their navigation. The path integration with odometry information is related to calculating the accurate home location [11,12,13,14,15]. Cumulative errors in path integration can be compensated by visual cues including the skyline and polarized information, as well as the surrounding landmark information [16,17,18]. There are many other examples with visual cues [19,20,21,22], and also, their navigation involves many types of sensors including vision, olfactory, auditory, odometry and magnetic sensors. [23,24,25,26]

**Figure 1:**
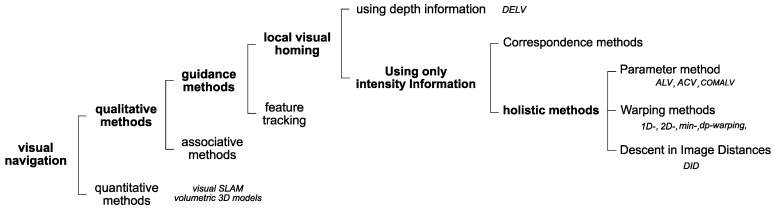
Overview of visual navigation. DELV, Distance Estimated Landmark Vector; ALV, Average Landmark Vector; ACV, Average Correctional Vector.

There have been many local visual homing techniques. We can largely divide these into two parts using depth or intensity information [27] as shown in Figure 1. Furthermore, intensity-based approaches can be divided into holistic methods and correspondence methods. Correspondence methods match extracted features in the images and thus need a complex algorithm to process feature extraction and feature matching. If there is a reference compass available, the one-to-one correspondence matching can be omitted. Holistic methods try to match the whole pixel information in the two images without extracting landmark features or classifying the features. Holistic methods have relatively low complexity.

Landmark vector models to represent the surrounding environment have been studied, and the models often use angular positions of landmarks without landmark matching, if a reference compass such as a light or magnetic compass is available and the two snapshots can be aligned with the reference coordinate. The Average Landmark Vector (ALV) model [28] is a typical example of the landmark vector model covering the omnidirectional view. Even if it is assumed that all the landmarks on the retinal image have equal distances, the whole distribution of landmarks can be simply represented as the average landmark vector, each of which has a unit length with its angular position. According to the snapshot model [10], the model compares two ALVs obtained from the home snapshot and the current view. The difference between the two vectors can estimate the homing direction from the current position. The ALV model can be combined with visual feature detection [29]. The Average Correctional Vector (ACV) [30,31] is a variation of the ALV model. This model uses the amount of angular differences as the length of the landmark vector. Another method, the Distance Estimated Landmark Vector (DELV) model [32,33], suggests encoding distance in the landmark vectors and provides better estimation of the homing vector at the current position by localizing the current position in a reference map. The snapshot matching method can be combined with optic flow [34], and it has been applied to aircraft trying to estimate the current location.

In the holistic methods, there have been two ways to handle the whole image pixels, the image distance model and the warping model. The Descent in Image Distance (DID) method [35] uses the all pixels in a pair of images to calculate the image distance. The pixel difference between the snapshot image at a given position and the home snapshot can roughly estimate the relative distance between the two positions. If a snapshot among candidate snapshots at different positions is close to the home snapshot in terms of the image distance, it is assumed that the direction to the snapshot is close to the homing direction. More advanced models following the idea have been studied [36,37]. Homing in scale space [38] uses correspondences between SIFT features and analyzes the resulting flow field to determine the movement direction. It is mathematically justified by another study [39]. Another view-based homing method based on the Image Coordinates Extrapolation (ICE) algorithm has been tested [40].

In contrast to the above image distance models, there have been warping methods to calculate all possible matchings between all pixels in a pair of images as another holistic approach. In the one-dimensional warping model [41,42,43], all possible changes of pixels along the horizontal line are calculated under the assumption that landmarks have equal distances. The homing direction can be estimated by searching for the smallest difference in a particular angle between the candidate image and the home reference image. There are also advanced warping models including the 2D-warping and min-warping model [44,45,46] that apply a variation of alignment angle estimation for the environment without a reference compass. There have been other variations of the warping model [47,48]. Recently, a method with various visual features like SURF has been compared with the holistic approach for robotic experiment [49]. Generally, the holistic methods show robust performance, but need high computing time.

The image warping methods normally have an equal distance assumption for landmarks. However, the visual information is easily changed by position, distance, luminous sources, shades or other environmental factors. They can produce large homing error depending on the environmental situation. The distance information has been very often neglected in the holistic approaches, as well as landmark vector models, although it can contribute to reading the surrounding environmental information. Recently, it was shown that the depth information greatly improves the homing performance [33,50]. In this paper, we suggest a moment model to combine the distance information with visual features or image pixel information.

Another issue in local visual homing is related to an alignment problem of two snapshot images. If there is no reference compass, the orientations of the current view and the home need to be aligned together. That is, one visual feature in the current view should correspond to a visual feature in the home snapshot, since the visual feature points to the same landmark object. A solution to match the two different orientations is to calculate the image differences between the home snapshot and the rotated image of the current snapshot and find the rotation angle of the current view with the minimal image difference, which is called the visual compass approach [35]. Another approach is the landmark arrangement method [51] in which a set of visual landmarks in the current view are re-mapped to visual landmarks one by one in the reference coordinate, and a circular shift of the landmarks is applied repeatedly to find the best matching of the visual landmarks in the two orientations by checking if a set of the resultant homing vectors starting from each landmark has converged into one point with small variance. In our experiments, we will test the above two approaches for the alignment of the two snapshot images, if there is no reference compass.

In the complex cluttered environment, the Simultaneous Localization and Mapping (SLAM) method has been popular. SLAM often uses a laser sensor for distance information to build a map for the environment. Interestingly, a moment model called Elevation Moment of Inertia (EMOI) has been suggested to imitate a physical quantity, moment of inertia [52]. The model characterizes the environmental landscape, the surrounding range value and height information as a scalar value called EMOI. Inspired by the model, we suggest a new type of moment function to cover various environmental information, which can read the landmark distribution from the environment with two components, the distance to landmarks and the visual feature of landmarks.

The main contribution of our work is to suggest a new type of moment potential to guide homing navigation and prove the convergence of the moment model to a unique reference point. The homing navigation follows the snapshot theory to compare two snapshots to determine the homing direction. We provide a homing vector estimation based on the reference point in the moment model for a pair of snapshots. Furthermore, it is shown that the moment model can encode the landmark distribution and features. Our approach can be extended to a moment model with multiple features, which can produce multiple reference points for robust homing performance. The combinational model with distance and visual features shows better homing performance than the distance information alone or the visual features alone. We demonstrate robotic homing experiments with the moment model and various methods.

## 2. Method

### 2.1. Robot Platform and the Environment

**Figure 2:**
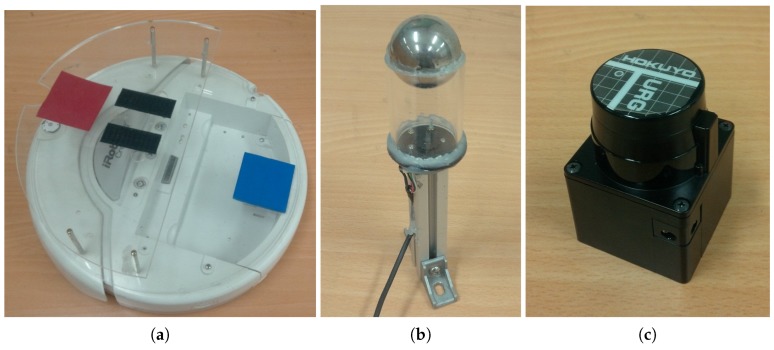
Mobile robot platform: (**a**) i-Robot ROOMBA mobile; (**b**) omnidirectional camera; (**c**) HOKUYO laser sensor.

**Figure 3:**
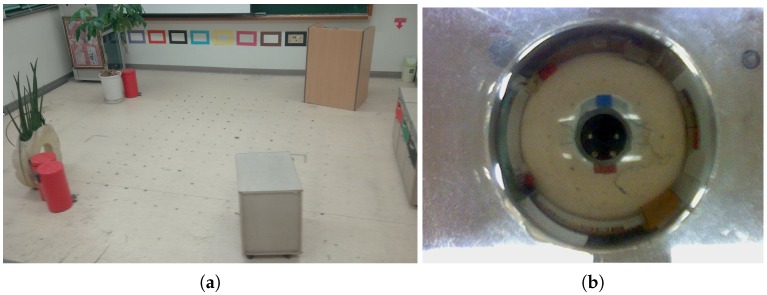

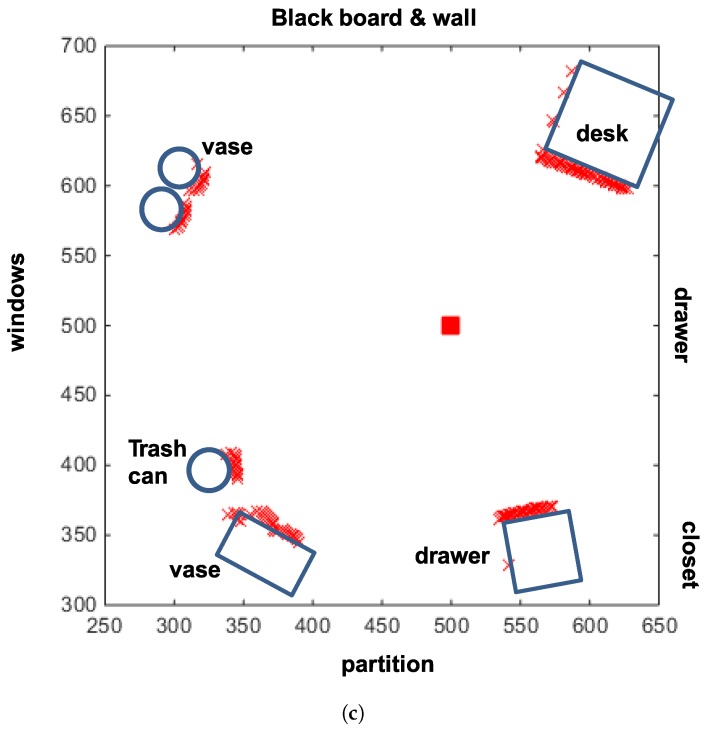
Experimental environment *env*0: (**a**) a view of the environment; (**b**) omnidirectional image from the camera; (**c**) distance map from the range sensor; the red square indicates the home position (500,500).

We test robotic experiments in a 6 m × 6 m room with several objects including dresser, drawers, a trash can, a plant, windows and walls. We use i-Robot Roomba for a mobile robot with two wheels, which is connected to a laptop computer for control. Here, the robot platform can read a panoramic image of the surrounding environment through an omnidirectional camera in Figure 2, which consists of a Logitech Webcam E3500, a metal ball for a reflection mirror and the acryl support for mounting. The robot can also be mounted with a HOKUYO laser sensor URG-04 LX model shown in Figure 2. The laser sensor can cover a range of 240 degrees in angular space with 0.36 degree resolution. To obtain an omnidirectional distance image, two shots from the laser sensor are needed. In this way, we can collect both an omnidirectional color image and depth information. Figure 3 shows an example of the reconstructed image and depth map for the surrounding environment (red × marks show sensor readings from the laser sensor).

**Figure 4:**
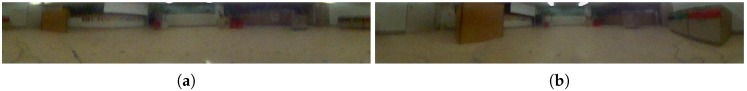

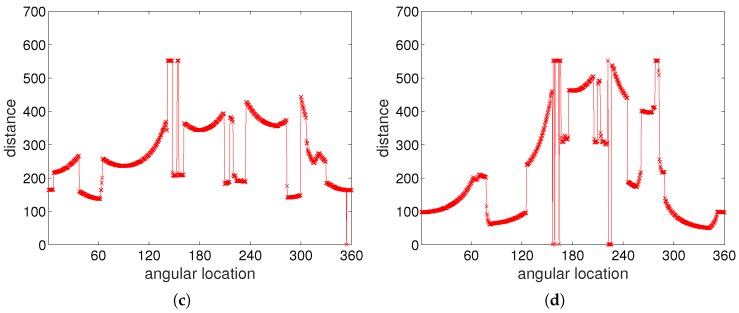
Omnidirectional image and distance map: (**a**,**b**) panoramic images at (50,50) and (62,54), respectively; (**c**,**d**) omnidirectional depth information at at (50,50) and (62,54), respectively.

An omni-directional image that the robot takes has 640 × 480 RGB pixels. It is converted into a panoramic image, 720 × 120 pixels (with 0.5 degree angular resolution), with a uniform size of pixels for each angular position, while the omnidirectional image has a relatively small number of pixels near the observation point. The panoramic form can easily access the pixel in terms of the angular position and distance, the angular position in the *x*-axis and distance from the center of the image in the *y*-axis. Figure 4a,b shows examples of panoramic images obtained at two different positions. Figure 4c,d shows range data from laser sensor readings corresponding to the panoramic images. The snapshot images or snapshot distance maps have a similar landscape, but they are distorted depending on the position.

### 2.2. Moment Model for Landmark Distribution

In physics, the moment of inertia is a property of an area that reflects how its points are distributed. By analogy, the moment is defined as a distribution of point measurements in our navigation model.

For a given set of landmarks in the environment, we analyze the landmark distribution as a combination of their positions and features. The color intensity or height of landmarks can be feature candidates. A landmark is defined as a natural feature in the world environment, which is observable even at a far distance. All the landmarks are projected into the image plane, and the snapshot view includes a collection of landmarks. Often, an object is represented with a cluster of pixels in the image through the image segmentation process. Without any object feature extraction, each pixel in the image view can be regarded as a landmark, and then, the feature extraction process can be omitted. In real environments, the color pixels in the surrounding view (omnidirectional view) are regarded as landmarks with the color intensity, as well as the range information. Even the background at a far distance is represented as a set of landmarks. If salient landmarks are identified from the background, only those landmarks may be used in our moment model.

The color of the visual cue is the feature used in this paper. We define the moment measure *M* as follows:

(1) $$M=\sum_{i=1}^{N}{r_{i}}^{2}C_{i}=\sum_{i=1}^{N}\left({\left(x-a_{i}\right)}^{2}+{\left(y-b_{i}\right)}^{2}\right)C_{i}$$

where there are *N* landmarks, $r_{i}$ is the range value of the *i*-th landmark, that is the distance from the current location (x,y) to the landmark location $\left(a_{i},b_{i}\right)$, and $C_{i}$ is the feature value, for example the color intensity of the *i*-th landmark.

The above measure is similar to the moment of inertia in physics, $\int r^{2}dm$. We can also see this measure as a potential function built with a set of landmarks. From that, we can find the gradient as the first derivative of the potential function as follows:

(2) $$\nabla M=\left(\frac{dM}{dx},\frac{dM}{dy}\right)=\left(M_{x},M_{y}\right)=\sum_{i=1}^{N}(2\left(x-a_{i}\right)C_{i},\;\;\;2\left(y-b_{i}\right)C_{i})$$

where this gradient vector indicates the change of the potential function corresponding to the current position (x,y). To find the minimum convergence point with the gradient, we calculate the determinant of the Hessian matrix given below:

(3) $$H=\left(\begin{array}{cc} \frac{d^{2}M}{dx^{2}} & \frac{d^{2}M}{dxdy}\\ \frac{d^{2}M}{dxdy} & \frac{d^{2}M}{dy^{2}} \end{array}\right)=\left(\begin{array}{cc} M_{xx} & M_{xy}\\ M_{yx} & M_{yy} \end{array}\right)=\sum_{i=1}^{N}\left(\begin{array}{cc} 2C_{i} & 0\\ 0 & 2C_{i} \end{array}\right)$$

where this Hessian matrix is produced from the second-order differential of the moment equation. $M_{xx}$ is a second-order differential with respect to *x* and $M_{yy}$ with respect to *y*. $M_{xy}$ is equal to $M_{yx}$, and they are zero.

The determinant of the Hessian matrix is calculated as:

(4) $$\det\left(J\right)=\left(\sum_{i=1}^{N}2C_{i}\right)\times \left(\sum_{i=1}^{N}2C_{i}\right)={\left(\sum_{i=1}^{N}2C_{i}\right)}^{2}>0$$

We assume that each feature value ($C_{i}$) is positive. The sign of the second derivatives of potential function is positive as shown below:

(5) $$M_{xx}=M_{yy}=\sum_{i=1}^{N}2C_{i}>0$$

From the above property, there is only one global convergence (minimum potential) point with its gradient zero, and the determinant of the Hessian matrix is positive. Let (X,Y) be the convergence point. Then:

(6) $$\nabla M\left(X,Y\right)=\sum_{i=1}^{N}(2\left(X-a_{i}\right)C_{i},\;2\left(Y-b_{i}\right)C_{i})=0$$

The position (X,Y) is calculated as:

(7) $$X=\sum_{i=1}^{N}a_{i}C_{i}/\sum_{i=1}^{N}C_{i},\;\;\;\;\;Y=\sum_{i=1}^{N}b_{i}C_{i}/\sum_{i=1}^{N}C_{i}$$

where the convergence point (X,Y) is the weighted average of landmark positions with respect to the landmark features, that is the center of the landmark distribution. The moment measure based on features of landmarks has unique convergence point (X,Y), regardless of any current position (x,y).

**Figure 5:**
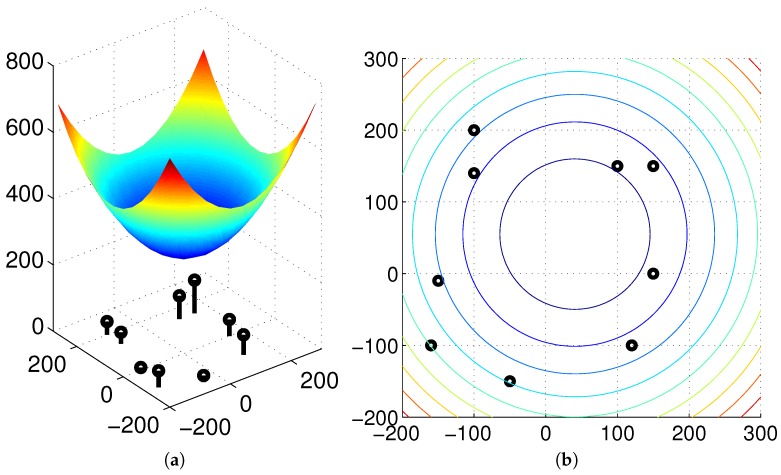
Moment measure as a potential function where the landmark height is regarded as a feature value: (**a**) potential function, (**b**) contour line.

Thus, we argue that if there is no environmental change or no occlusion observed as the robot moves, then we can find the same convergence point in spite of any movement or any change in angular position. To guarantee the unique convergence point, the feature value should be positive. In our experiments, the landmark characteristics are defined as the height of landmarks or color intensity, which is positive. The moment measure is an index of landmark distribution, and its center of distribution can easily be estimated as an invariant feature, which will be useful in homing navigation. In Figure 5, the surface of the potential function is convex-shaped, and the unique convergence point is available. Various types of feature ($C_{i}$) values are available, and the convergence point can change depending on the features.

### 2.3. Homing Vector Using the Moment Model

We introduce how to estimate the homing vector using the moment function. We assume there is a reference compass available. Each landmark has the feature value and range information. An agent can observe a distribution of landmarks at a given position. Assume the same landmarks are observed at any position in the environment. We take the above global convergence point as the reference point to estimate the homing vector.

If there are *N* landmarks observed at the current position P=(x,y), their relative distance $r_{i}=\left|\right|\left(a_{i}-x,b_{i}-y\right)\left|\right|$ and the feature value $C_{i}$ are measured for i=1,…,N, where $\left(a_{i}-x,b_{i}-y\right)$ is the estimated landmark position in the coordinate with origin at the current position. The reference point vector $R=(X_{c},Y_{c})$ can be calculated by Equation (7). In a similar way, *N* landmarks are observed at the home position $\bar{H}=\left(H_{x},H_{y}\right)$. Their relative distance $r_{i}^{\prime }=\left|\right|\left(a_{i}^{\prime }-H_{x},b_{i}^{\prime }-H_{y}\right)\left|\right|$ and the feature value $C_{i}^{\prime }$ are measured for i=1,…,N, where $\left(a_{i}^{\prime }-H_{x},b_{i}^{\prime }-H_{y}\right)$ is the estimated landmark position with origin at the home position $\bar{H}$. The reference point vector $R^{\prime }=\left(X_{h},Y_{h}\right)$ can be calculated by Equation (7) again at the home position.

Then, we find the relation for homing vector $\vec{H}$:

(8) $$\vec{H}=\bar{H}-P≃R-R^{\prime },$$

since we assume that the same reference point is estimated irrespective of any observation point, that is the two vectors *R* and $R^{\prime }$ should end at the same reference point, starting from the different positions, the current position and the home location (a little deviation of the reference points may be observed by noisy sensor readings or landmark occlusions).

At an arbitrary position P=(x,y), a mobile robot has information of the relative distance and the visual features with a laser sensor and a vision camera. Equation (7) has absolute coordinate representation, and so, we evaluate the convergence point in the coordinate with origin at the observation point.

(9) $$X_{c}=\sum_{i=1}^{N}\left(a_{i}-x\right)C_{i}/\sum_{i=1}^{N}C_{i},\;\;\;\;\;Y_{c}=\sum_{i=1}^{N}\left(b_{i}-y\right)C_{i}/\sum_{i=1}^{N}C_{i}$$

where P=(x,y) is the current observation point and $\left(a_{i}-x,b_{i}-y\right)$ is the relative distance of the *i*-th landmark in the current view. Similarly, the convergence point can be evaluated in the home coordinate as follows:

(10) $$X_{h}=\sum_{i=1}^{N}\left(a_{i}^{\prime }-H_{x}\right)C_{i}^{\prime }/\sum_{i=1}^{N}C_{i}^{\prime },\;\;\;\;\;Y_{h}=\sum_{i=1}^{N}\left(b_{i}^{\prime }-H_{y}\right)C_{i}^{\prime }/\sum_{i=1}^{N}C_{i}^{\prime }$$

where $\left(H_{x},H_{y}\right)$ is the home location. Then, the difference of the two reference points measured at two observation points (the home location and the current position) is given by:

(11) $$\begin{array}{ccc} R-R^{\prime } & = & \left(X_{c},Y_{c}\right)-\left(X_{h},Y_{h}\right) \end{array}$$

(12) $$\begin{array}{cc} = & \left(\frac{\sum \left(a_{i}-x\right)C_{i}}{\sum C_{i}},\frac{\sum \left(a_{i}-y\right)C_{i}}{\sum C_{i}}\right)-\left(\frac{\sum \left(a_{i}^{\prime }-H_{x}\right)C_{i}^{\prime }}{\sum C_{i}^{\prime }},\frac{\sum \left(a_{i}^{\prime }-H_{y}\right)C_{i}^{\prime }}{\sum C_{i}^{\prime }}\right) \end{array}$$

(13) $$\begin{array}{cc} ≃ & \left(\frac{\sum \left(a_{i}-x\right)C_{i}}{\sum C_{i}},\frac{\sum \left(a_{i}-y\right)C_{i}}{\sum C_{i}}\right)-\left(\frac{\sum \left(a_{i}-H_{x}\right)C_{i}}{\sum C_{i}},\frac{\sum \left(a_{i}-H_{y}\right)C_{i}}{\sum C_{i}}\right) \end{array}$$

(14) $$\begin{array}{cc} = & \left(\frac{\sum \left(H_{x}-x\right)C_{i}}{\sum C_{i}},\frac{\sum H_{y}-y)C_{i}}{\sum C_{i}}\right) \end{array}$$

(15) $$\begin{array}{cc} = & \left(H_{x}-x,H_{y}-y\right)=\bar{H}-P \end{array}$$

where it is assumed that the same landmarks and the same visual features are observed at any position, $\left(a_{i},b_{i}\right)=\left(a_{i}^{\prime },b_{i}^{\prime }\right)$, $C_{i}=C_{i}^{\prime }$ for i=1,…,N. Hence, the homing vector $\vec{H}$ can be estimated by the above property,

(16) $$\vec{H}=\left(X_{c},Y_{c}\right)-\left(X_{h},Y_{h}\right)$$

Each position defines its own reference map, but there exists a unique convergence point that is the same position regardless of any coordinate. By the convexity of the moment potential function, the minimal potential point can be reached from any position. We provided a proof that the homing vector calculated by the above model can reach the home position from any position, if the environment is isotropic, that is all landmarks and their features are invariantly observed at any position.

### 2.4. Moment Model with Multiple Features

If there are multiple features available for landmarks, then we can build a separate moment model for each feature. The set of moment models will lead to independent reference points, but we can assume that the distribution of each feature in the environment will be almost equal for any measured position if the environment is isotropic, that is the majority of landmarks are commonly observed in the environment. The homing vector for each feature can be voted together, which can help estimate homing direction more accurately.

We can test the moment model with RGB color intensities, three visual features for each pixel. The image colors provide three different features, red, green and blue color intensity for each pixel. The landmark feature $C_{i}$ can thus have three components. The moment measure for each feature, red, blue and green intensity, respectively, is defined as follows:

(17) $$M_{R}=\sum_{i=1}^{N}{r_{i}}^{2}R_{i}=\sum_{i=1}^{N}\left({\left(x-a_{i}\right)}^{2}+{\left(y-b_{i}\right)}^{2}\right)R_{i}$$

(18) $$M_{G}=\sum_{i=1}^{N}{r_{i}}^{2}G_{i}=\sum_{i=1}^{N}\left({\left(x-a_{i}\right)}^{2}+{\left(y-b_{i}\right)}^{2}\right)G_{i}$$

(19) $$M_{B}=\sum_{i=1}^{N}{r_{i}}^{2}B_{i}=\sum_{i=1}^{N}\left({\left(x-a_{i}\right)}^{2}+{\left(y-b_{i}\right)}^{2}\right)B_{i}$$

where $\left(a_{i},b_{i}\right)$ for i=1,…,N are the landmark position with respect to the current position P=(x,y) and $\left(R_{i},B_{i},G_{i}\right)$ are the color intensity for the *i*-th landmark.

Then, the above three measures lead to three reference points at a given position P=(x,y), using Equation (9).

(20) $$(X_{R},Y_{R})=\left(\frac{\sum \left(a_{i}-x\right)R_{i}}{\sum R_{i}},\frac{\sum \left(b_{i}-y\right)R_{i}}{\sum R_{i}}\right),$$

(21) $$(X_{G},Y_{G})=\left(\frac{\sum \left(a_{i}-x\right)G_{i}}{\sum G_{i}},\frac{\sum \left(b_{i}-y\right)G_{i}}{\sum G_{i}}\right),$$

(22) $$(X_{B},Y_{B})=\left(\frac{\sum \left(a_{i}-x\right)B_{i}}{\sum B_{i}},\frac{\sum \left(b_{i}-y\right)B_{i}}{\sum B_{i}}\right),$$

Three reference points can be determined both at the current position and at the home location. The difference of the reference points can estimate the homing direction.

The home vector $\vec{H}$ via the three reference points using the color intensities can be derived as a combinational form,

(23) $$\vec{H}=\left(\vec{H_{R}}+\vec{H_{G}}+\vec{H_{B}}\right)/3$$

where $\vec{H_{R}}$ is calculated with red color intensity, $\vec{H_{G}}$ with green and $\vec{H_{B}}$ with blue, using Equation (16).

As shown above, the color intensity of pixels can be applied to the moment model with multiple features. We can extend the moment measure into that with various visual features. The visual feature $C_{i}$ allows any characteristics of landmarks, and also, multiple features can derive multiple homing vectors. The sum of the homing vector for each feature can be effective on noisy feature readings. RGB color space can be converted into another space, for example HSV space, and each feature can make separate homing vectors. Furthermore, to handle noisy sensor readings, we can allow a cut-off threshold for a feature value, and some feature can be set to $C_{i}=0$. This has the effect of choosing a set of landmarks in the omnidirectional view, instead of using the whole pixels. If $C_{i}=0$ or $C_{i}=1$ with the range value $r_{i}=1$, the moment model becomes similar to the ALV model [28]. If $C_{i}=0$ or $C_{i}=1$ with continuous range value $r_{i}>0$, the model is similar to the DELV model [33].

### 2.5. Comparison with Other Methods

To compare our moment model with other conventional approaches, we consider possible combinations of the process components. The components are related to what kind of features will be used, whether the range sensor is available or the distance can be estimated in the visual image and what kind of coordinate alignment process will be applied.

In the moment model, we can allow variable features in $C_{i}$. In our experiments, we will mostly use the RGB color intensity as the feature value. To compare variable visual features and no visual feature, we can test $C_{i}=1$ for equal color intensity. In addition, the moment model requires the distance information to calculate the centroid. Since there is no distance information of landmarks in the image set, we estimated the distance using the ground line in the image, which was used for the moment model. The ground line is the boundary line between the floor and an object in the panoramic image. After blurring the whole image, a moving mask (4 × 2 pixels) to detect the horizontal edge was applied, and the number of pixels between the horizontal line and the detected ground line was counted to estimate the distance; more pixels counted indicate a larger distance to the ground or the landmark. We discriminate the methods with the ground-distance estimation in the visual image and those with the laser sensor readings for distance.

Without a reference compass, the snapshot images at two positions are not aligned. Their image coordinates may be different. We need to make an effort to find the rotation angle of a given coordinate to be matched to another coordinate. One of the well-known algorithms for alignment is the visual compass [35]. The home snapshot is taken as a reference image, and the current view is shifted a step angle until the home image and the shifted image have the minimum difference. The shifted angle is the right angle in the coordinate alignment.

Another alignment algorithm called landmark rearrangement is available [51]. A set of landmarks observed in the home coordinate should match another set of landmarks observed at the current position, if it is assumed that the environment is isotropic. That is, two sets of landmarks at the home location and at the current position should correspond to each other. If two coordinates (orientations) are not aligned, then the two sets of landmarks should be compared for one-to-one correspondence by a rotating shift of one landmark at a time until they closely match each other. The landmark rearrangement method first draws landmark vectors from the home location to each landmark and then adds the opposite of landmark vectors from the current position to each landmark by one-to-one mapping. Then, the sum of the resulting vectors can be converged to a point, if there is no matching error. If the two coordinates are not aligned and landmarks have a mismatch, the end points of the resulting vectors have a large variance. We use this variance criterion to make the two coordinates aligned by following the landmark rearrangement [51]. We need to apply a rotational shift of landmarks in a given coordinate to update a series of landmarks.

For visual homing navigation, the Descent in Image Distance (DID) method [35] can be applied to calculate homing direction, which uses snapshots near the current position and the home snapshot. We take a variation of the DID with multiple (three) images as reference images and the snapshot at the current location in order to determine the homing direction. Using the property that the absolute image difference between a pair of snapshots is proportional to the distance, the snapshot at the current position is compared with three snapshots (including the home snapshot) near the home location, and then, the image differences can determine the homing direction by the relative ratio of the image distance between each of the three snapshots and the current image. This DID method uses only visual images, and the relative difference of the in-between images can guide the homing direction without any depth sensor.

Table 1 shows various methods classified by the feature selection, the range sensor or distance estimation and the alignment process. With the reference compass, no alignment process is required, and six methods are available. Without a reference compass, eight methods are listed (for $C_{i}=1$, no visual feature is observed and only landmark rearrangement will be tested, since the visual compass is not applicable).

## 3. Experimental Results

We defined the moment function as a property to characterize the landmark distribution consisting of the distance and visual features. Initially, we will see the homing performance with the model in the simulation environments. Then, we will test the moment model for homing navigation in the indoor environments. We also investigate the model under various conditions to see the effect of the visual features, the landmark distance and the coordinate alignment (orientation alignment).

### 3.1. Simulation Environment

**Figure 6:**
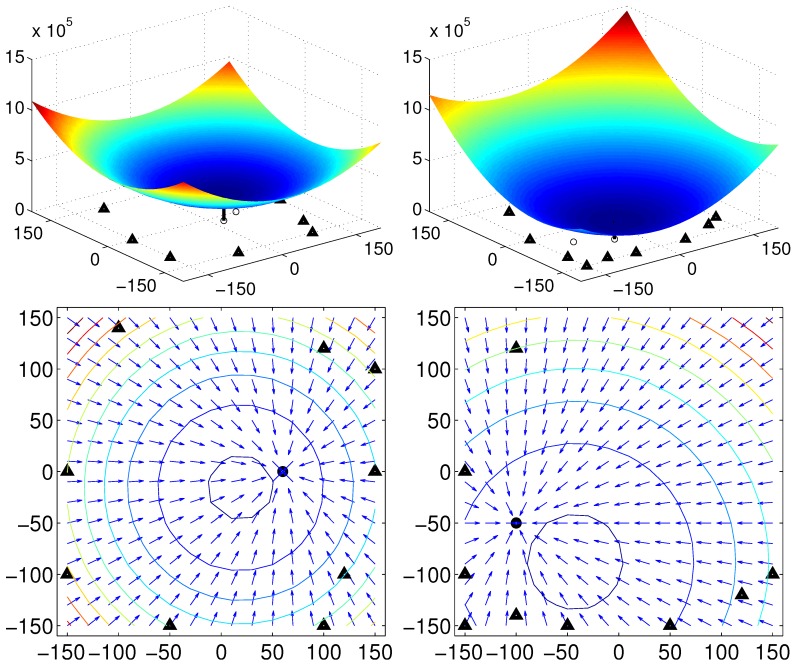
Test with two different simulation environments; the first row shows the moment potential, and the second row shows vector maps including the homing vector on the contour plot (triangles: landmark positions, black dot: home location).

Initially, we test our approach in the simulation environment consisting of a set of salient landmarks instead of pixel-wise landmarks. In the environment, it is assumed that an agent can observe extrusive objects discriminated from the background area at a far distance, and those objects are regarded as landmarks. Furthermore, the agent sees all the landmarks at any position. A set of landmarks (discrete landmarks) is given in an arena, and each landmark has its height. We assume that the distance to each landmark is obtained with a laser sensor. In Figure 6, we can observe the unique convergence point with a feature value of height for each environment. The home location may be different from the convergence point (with the minimum potential), but the moment model guides the homing direction well. In the simulation environment, a mobile agent makes no obstacle avoidance behavior and only moves towards the target position, assuming all the object landmarks are visible at any position. Figure 6 shows only homing vectors calculated at a grid position based on the moment model by ignoring the obstacles.

Figure 6 shows that the moment potential has a convex shape and it has the minimal peak point. Even if the landmarks are not uniformly distributed, the moment measure has the minimal reference point. A pair of positions including the home location can share the same reference point using the relative distances to a set of landmarks and the landmark features. In the simulation environment, there is no error to estimate the reference point, and the homing direction at each position is very accurate.

### 3.2. Moment Model in Real Environments with Depth Sensor Readings

We investigate the possibility of the moment model in real environments. The moment measure allows various features for landmarks in its calculation. In our experiments, a holistic approach over the landmark distribution is used, and the omnidirectional depth information in combination with visual features are encoded in the moment value.

**Figure 7:**
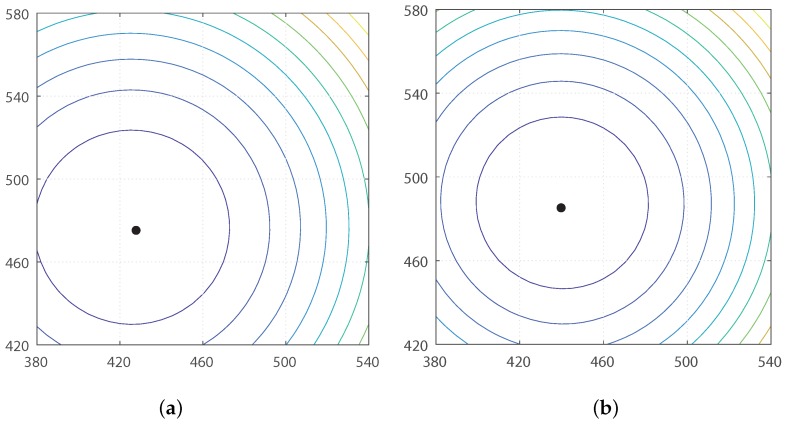
Contour plots at two different positions in the indoor environment env0 (Figure 3). (**a**) Contour of moment potential at (460,460); (**b**) contour of moment potential at home position (500,500); black dots indicate the minimum potential points.

Initially, we measured the moment with RGB color intensities in the panoramic image. The holistic view takes all the pixels as landmarks in the environment. The information about 720 pixels near the horizontal line and the corresponding depth determines a moment potential. The contour map of the moment potential is displayed in Figure 7. The range sensor readings and color intensities can change depending on the observation point. Figure 7 shows that the minimal potential positions closely match each other in the real environment, although there is a little deviation of the minimal potential position (reference point) when it is calculated at two different observation points. The measurement error or a few occlusions of landmarks can induce a small deviation of the ideal reference point, thus causing homing error potentially.

**Figure 8:**
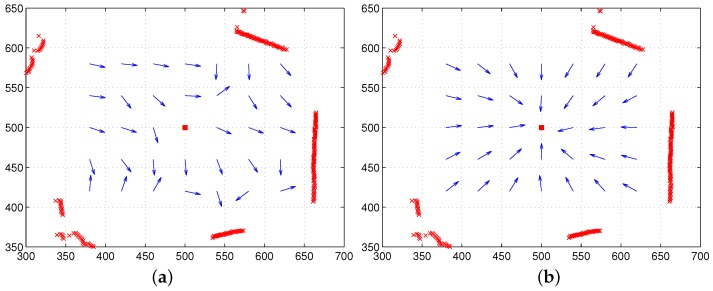
Homing performance only with color intensity or only with range data (reference compass available): (**a**) only with vision, but the unit distance ($r_{i}=1$) (all landmarks have equal distances); (**b**) with only range data, but the unit feature ($C_{i}=1$) (all landmarks have the same visual feature or same color intensity) (the red box at (500,500) indicates home, and arrows show the homing direction at each point).

To see the effect of the two components, the color intensity and the distance of landmarks, we tested the moment model with the unit distance $r_{i}=1$ or the unit feature $C_{i}=1$. In the first test, only color intensity is available for landmarks under the equal distance assumption (unit distance $r_{i}=1$). Figure 8a shows the result, and relatively large homing errors are observed. This implies that landmarks only with color intensity have difficulty in representing the surrounding environment. The distribution of RGB colors changes depending on the observation point, which will highly influence the estimation of the reference point, as well as the homing direction. In contrast, another test with continuous-ranged distance, but no color intensity shows much better homing performance; see Figure 8b. The depth information of landmarks greatly contributes to the estimation of the homing direction.

**Figure 9:**
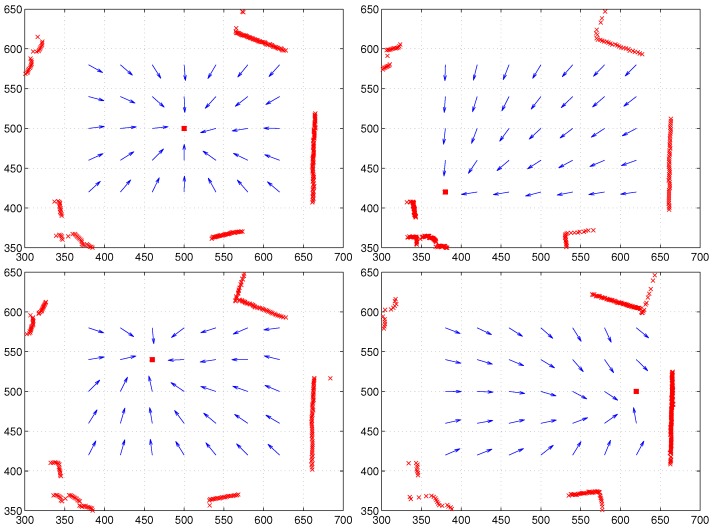
Homing performance with both range data and color intensity (reference compass available); the red box indicates the home position, and the arrows show the homing direction at each point.

Figure 9 shows vector maps using both the distance information and color intensity when a reference compass is available. The moment model with both features shows good homing performance at any position in the environment, mostly better than the moment model only with distance. Thus, the moment model, a combination model of distance and visual features, is a more promising approach to read the landmark distribution or characteristics in the environment.

**Figure 10:**
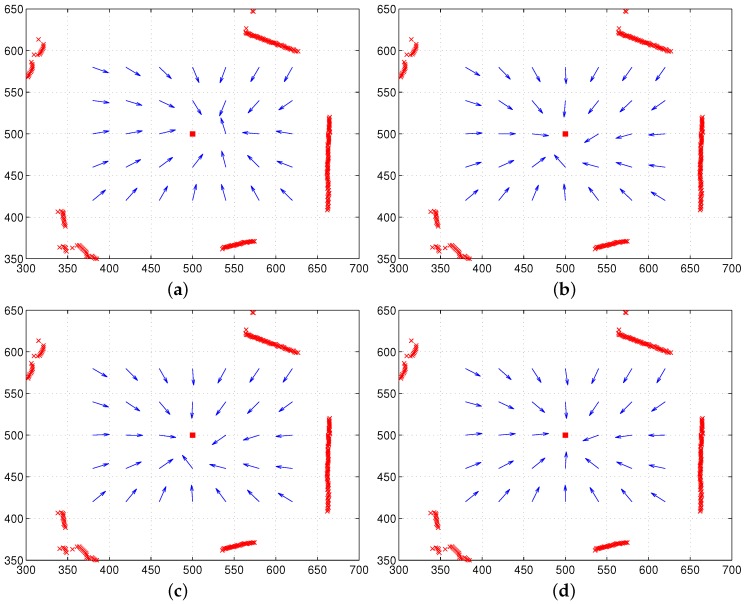
Homing performance with various feature values: (**a**) $C_{i}$ is the blue color intensity; (**b**) $C_{i}$ is the red color intensity; (**c**) $C_{i}$ is the V intensity in HSV space; (**d**) $C_{i}=1$ or 0 depending on the discretized condition with HSV (if $\sqrt{H^{2}+S^{2}+V^{2}}>50$, $C_{i}=1$, otherwise $C_{i}=0$); the red box at (500,500) indicates home, and arrows show the homing direction at each point.

Instead of the RGB space, we can apply one color, for example red-colored intensity, to the moment measure. In this case, a single reference point is available to derive the homing vector. Furthermore, the HSV space for one pixel can produce another visual feature, and the moment with the HSV intensity can derive different reference points; but it also produces successful homing performance. To discretize the continuous-ranged attribute value, a cut-off threshold can be used; if a feature value is greater than the threshold, we set $C_{i}=1$, otherwise $C_{i}=0$. In this case, the moment measure is like extracting a special feature of landmarks, by collecting all the landmarks with $C_{i}=1$. Then, the moment model can be converted to the ALV or the DELV model depending on the distance information (unit distance or continuous-ranged distance). Figure 10 shows the homing performance result depending on various patterns of the feature $C_{i}$. Even one color intensity together with depth information provides reasonable homing performance. More features or a different representation of the visual feature like the HSV space can estimate homing directions well. A choice of special features satisfying a given condition can also find homing directions; see Figure 10d.

**Figure 11:**
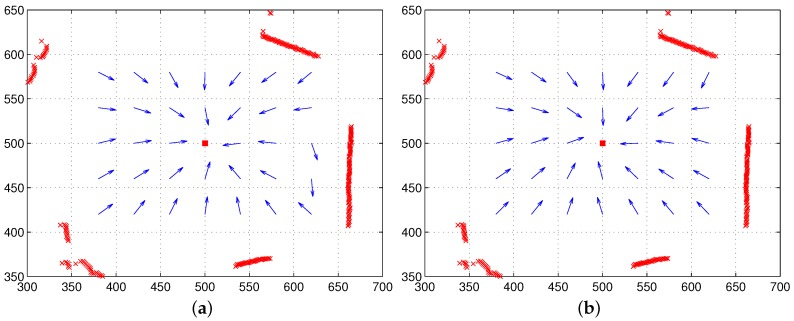
Homing performance of the moment model without a reference compass; both range data and color intensity are used, and the coordinate alignment process is applied: (**a**) visual compass; (**b**) landmark rearrangement method.

If there is no reference compass available, we need to align the coordinates for the snapshot images obtained at two positions. One of popular approaches is the visual compass approach by the pixel matching process. Another approach is the landmark rearrangement method. The methods were applied to the above environment. The moment model needs this alignment process a priori. Figure 11 shows the results. With the visual compass, there are some large angular errors at the right side corner, while the landmark rearrangement method shows relatively small angular errors towards home at all points.

**Figure 12:**
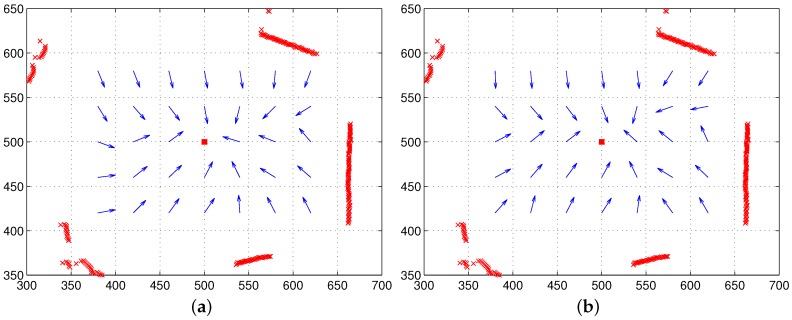
Homing performance with the DID approach (**a**) with a reference compass; (**b**) visual compass without a reference compass; the red box indicates the home position, and arrows show the homing direction at each point.

To compare the moment model and the DID (Descent in Image Distance) method, we demonstrated the homing results with the DID method in Figure 12. The DID method uses only visual images to estimate the homing direction. Despite the limitation, it can estimate the homing directions reasonably. It seems that whether the DID method has a reference compass or not has no large impact on the homing performance. Overall, the DID method has larger angular errors on average than the moment model, and this is due to lack of depth information of landmarks.

### 3.3. Moment Model in a Real Environment with Ground-Line Distance

We applied our moment model to another environment, one of Vardy’s dataset called ‘a1original’ [36,53], which includes many panoramic snapshots, but without a distance map. The omnidirectional images were collected for the indoor environment; the arena size is 2.7 m × 4.3 m, and there are 170 points with 10-cm regular intervals for snapshots.

**Figure 13:**
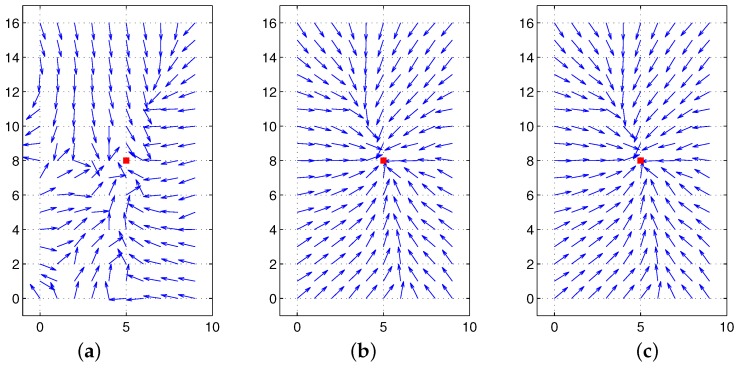
Homing performance for Vardy’s image environment with a reference compass: (**a**) moment model only with color intensity; (**b**) moment model only with estimated distance; (**c**) moment model with estimated distance and color intensity; distance is estimated using the ground line in the image without the laser sensor (the arrow indicates the homing direction).

Figure 13 shows the homing performance results with a reference compass. Similar to the previous results with our lab environment env0 shown in Figure 3, the moment model only with color intensity shows the worst performance. The model with ground-line distance shows very successful performance. The model with both distance and color intensity together shows a little more improved performance. Some positions have angular deviation in the direction to the goal position, which is related to the error in distance estimation. Without the laser sensor, only the visual image can determine the homing direction effectively. It can be inferred that the distance parameter in the moment model greatly influences the homing performance.

**Figure 14:**
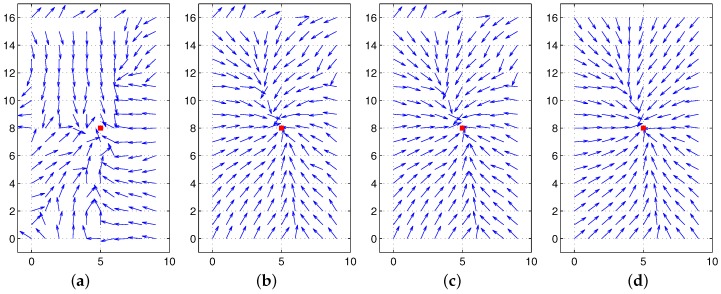
Homing performance for Vardy’s image environment without a reference compass: (**a**) visual compass and moment model only with color intensity; (**b**) visual compass and moment model only with estimated distance; (**c**) visual compass and moment model with both estimated distance and color intensity; (**d**) landmark rearrangement method and moment model with both estimated distance and color intensity; distance is estimated using the ground line in the image without a laser sensor (arrow indicates the homing direction).

**Figure 15:**
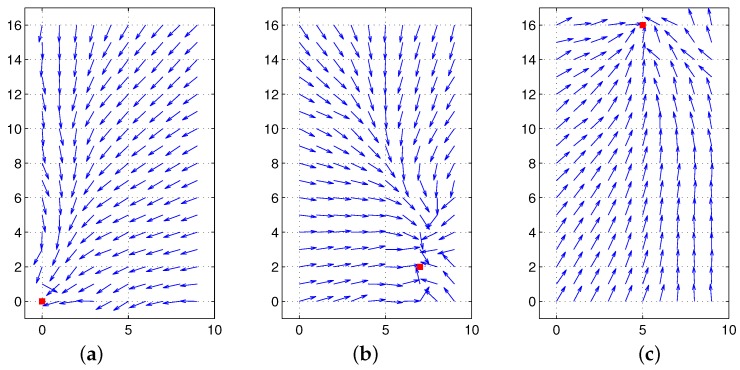
Homing performance for Vardy’s image set without a reference compass; the landmark rearrangement method to align the coordinate and the moment model with estimated distance and color intensity are used: (**a**) home location (0,0); (**b**) home location (7,2); (**c**) home location (5,16).

Figure 14 shows the performance depending on the coordinate alignment methods. Even without a reference compass, similar patterns of homing performance were observed. The moment model only with color intensity is insufficient to guide homing, but the model with the ground-line distance shows much better homing results. Furthermore, the landmark rearrangement method seems better than the visual compass method; see Figure 14c,d. Even when we change the home locations, the moment model with landmark rearrangement robustly works well for homing, as shown in Figure 15. With only visual snapshots, we are successful at estimating the distance to landmarks, as well as adjusting the coordinate alignment and deciding the homing direction ultimately.

### 3.4. Comparison of Homing Performances with Various Methods

We measured angular errors for homing directions with various methods listed in Table 1. We assume that the desired homing direction is the direct path from the current position to the home location. In the moment model, we encoded the depth information of landmarks and the visual features into a moment potential. We also tested the coordinate (orientation) alignment for the environment without a reference compass. The snapshot image at the home location becomes warped at the current position, and it influences the landmark distribution and the moment characteristics.

**Figure 16:**
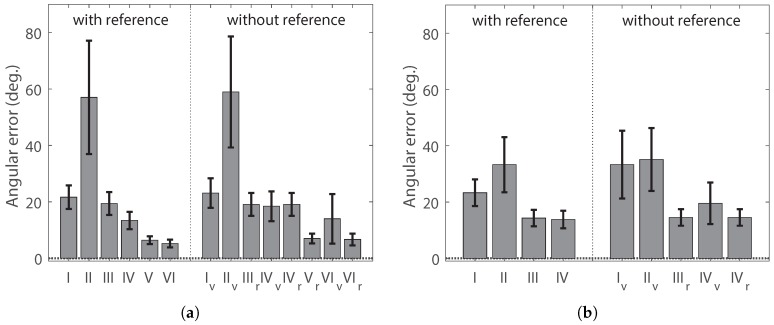
Homing performance with a reference compass and without a reference compass; the x-axis indicates testing methods described in Table 1: (**a**) angular errors in the indoor environment env0; (**b**) angular errors in Vardy’s image set; error bars indicate 95% confidence intervals by assuming a *t*-distribution.

The methods *I* and II use only visual features to decide the homing direction, and they have large angular errors. The moment model without depth information has shortcomings, compared to the DID method (the method *I*), as we see the performance for the methods *I* and II. However, in the moment model, the depth information of landmarks by a laser sensor or the estimation of the ground-line distance in the visual image significantly improves the homing performance; see the methods III–VI. The same result can be found in both the indoor environment *env*0 and Vardy’s dataset. Furthermore, if there is a real measurement of the distances to landmarks, it can further improve the performance as shown in Figure 16a.

In the moment model, the orientation alignment is required for the environment without a reference compass. We found that the landmark rearrangement is quite effective in aligning the coordinates (orientations). In many cases, the average performance with the landmark rearrangement is better than that of the visual compass, although not statistically significant. The methods (IV and VI) with a combination of two components, landmark distance and visual feature, have lower error performance, when compared to the methods with only distance (III and *V*). The DID method is based on the visual images and their image difference, and the above experiments imply that visual information alone seems insufficient to guide homing. The DID is significantly worse in homing performance than the moment model with the visual feature and the depth information.

Table 2 and Table 3 show angular errors for homing direction and 95% confidence intervals (assuming a *t*-distribution). Mostly, the errors are within 45 degrees. The method II with the visual feature alone shows a relatively large portion of angular errors greater than 45 degrees in the two different environments. The results are consistent with the performance shown in Figure 16. Especially, the DID method shows somewhat poor performance in Vardy’s image set without a reference compass.

## 4. Discussion

In the current approach, we used a holistic approach to take all the pixels as landmarks. Each pixel produces a landmark vector with its own angular position, instead of an object in the real environment. If an object is close to the observer, it creates many vectors, while a small number of vectors is assigned for an object far away from the observer. Summing these landmark vectors may be different from an object representation in the real environment. It is possible that the image segmentation or clustering process can help with identifying objects from the image. Then, the corresponding object features and the distances of objects in the environment can be encoded with the moment model. In other words, a sophisticated feature extraction algorithm can be combined with the suggested moment model. We need further work to check if this feature extraction approach will be better than the holistic approach. A basic assumption in this moment model is that invariant features can be observed at any position, and a collection of the features can represent the environment well, while its centroid can be localized as a reference point.

In our experiments, the RGB color intensity in the image view can be changed depending on the observation point. The visual images can be affected by the illumination, the angle and position of the light source, as well as glint on the floor. If these changes are intense, then the mobile robot cannot estimate the homing direction accurately. Thus, the robust features available or a good measurement apparatus will be helpful for homing accuracy. We believe that the range sensor has high accuracy of measuring the distance, but the visual image has relatively noisy sensor readings for pixel-wise landmarks. An ensemble of the two measurements seems to compensate one for the other. Possibly alternative features would be helpful to improve the homing performance. An invariant feature such as landmark height rather than the color intensity can be supportive to read the environmental information better, which can play the role of a milestone to guide homing.

The suggested moment function models a distribution of landmarks with their landmark characteristics. If we take a model with $M=\sum C_{i}/r_{i}$, the potential has a high peak at the landmark position, which can be used as a collision avoidance model or landmark search model. The potential value will rapidly decay to zero, if the measuring position becomes far from the landmark. If a mobile robot senses a potential value greater than a threshold, then it can take the behavior of avoiding the landmark or moving towards the landmark. This is another possible application of the moment model.

In our local homing navigation, we assume that landmarks are commonly observed at any position. The suggested moment approach is based on the snapshot model, which works only in the isotropic environment, and an agent can search for the target position as the home position, starting from the current position. Our local navigation approach can be extended to a wide range of navigation in complex cluttered environments with many occlusions of landmarks. Multiple reference images as milestones can guide the right direction to the goal position [37], even if the goal position is far way. That is, local homing navigation can be applied to each waypoint. A sequence of waypoint searches may lead to the final goal position. For future work, we will test this approach in cluttered environments to require complex homing paths.

## 5. Conclusions

In this paper, we suggest a new navigation method based on the moment model to characterize the landmark distribution and features. The moment model is inspired by the moment of inertia in physics, and it sees how the landmarks and their features are distributed in a given environment. Here, the landmark features and the relative distance are encoded in the moment function. The moment model allows multiple features, which can help estimate the homing direction more robustly. We proved that the landmark distribution has a unique minimum peak of moment potential, and it can be a reference point as an invariant feature at any position, which corresponds to the center of mass in physics. In the moment model, the homing vector is calculated based on this reference point.

In the experiments, we used the RGB color intensity in visual images as the features in the moment model. The distance was measured by a laser sensor, but without the depth sensor, the ground-line distance estimated in the panoramic image was tested as alternative distance information. The two components, the depth information and the features, highly contribute to successful homing performance, which is distinguished from other approaches’ results based on vision images. Especially, the depth information cannot be neglected for good homing performance.

Our approach is a holistic approach to use all the pixels as landmarks. We assumed that the environment is isotropic, that is the majority of landmarks is commonly observed in view at any position in the arena. If the mobile robot moves far away from the home location, the environment landscape will change greatly, and many occlusions occur, which may violate the isotropic assumption. In that case, the homing performance may degrade to a great extent, since the centroid of the moment value greatly migrates to another point. Thus, the moment model is appropriate for local homing navigation. We need further study to handle the problem or to cover a long-range path problem.

In the moment model, we simply applied the RGB color intensity as the visual feature. However, more sophisticated features, such as invariant features, can be developed in the image, which will further improve the homing performance. For future work, we will find what kind of visual features or other features will be useful in the moment model. Our model can be extended to various characteristics that are not easily changeable by the change of measuring positions. If the invariant property of the feature is preserved, the feature can be encoded in the moment model. Thus, various forms of the moment model can be produced.

## Figures and Tables

**Table 1:** Various methods classified by the feature, the range sensor and the alignment process.

	Method	Feature	Range	Alignment Method	Class
with reference compass	DID	color intensity	no sensor	aligned by compass	*I*
	moment	color intensity	equal distance ($r_{i}=1$)	aligned by compass	II
	moment	equal intensity ($C_{i}=1$)	ground-line estimation	aligned by compass	III
	moment	color intensity	ground-line estimation	aligned by compass	IV
	moment	equal intensity ($C_{i}=1$)	laser sensor	aligned by compass	*V*
	moment	color intensity	laser sensor	aligned by compass	VI
without reference compass	DID	color intensity	no sensor	visual compass	$I_{v}$
	moment	color intensity	equal distance ($r_{i}=1$)	visual compass	$II_{v}$
	moment	equal intensity ($C_{i}=1$)	ground-line estimation	landmark rearrangement	$III_{r}$
	moment	color intensity	ground-line estimation	visual compass	$IV_{v}$
	moment	color intensity	ground-line estimation	landmark rearrangement	$IV_{r}$
	moment	equal intensity ($C_{i}=1$)	laser sensor	landmark rearrangement	$V_{r}$
	moment	color intensity	laser sensor	visual compass	$VI_{v}$
	moment	color intensity	laser sensor	landmark rearrangement	$VI_{r}$

**Table 2:** Angular errors with various methods for the indoor environment *env*0; testing methods are described in Table 1 (*N* is the number of testing points, μ the average of angular errors, σ the 95% confidence interval (assuming a *t*-distribution)).

Method		error μ(±σ) deg.	N	$0\leq \varepsilon_{\theta }<45^{\circ }$	$45\leq \varepsilon_{\theta }<90^{\circ }$	$90\leq \varepsilon_{\theta }<180^{\circ }$
with reference compass	*I*	21 (±4.2)	34	97.1%	2.9%	0%
	II	57 (±20.1)	34	67.6%	5.9%	26.5%
	III	19.4 (±4.0)	34	97.1%	2.9%	0%
	IV	13.4 (±3.1)	34	100%	0%	0%
	*V*	6.4 (±1.4)	34	100%	0%	0%
	VI	5.7 (±1.4)	34	100%	0%	0%
without reference compass	$I_{v}$	23 (±5.2)	34	94.1%	5.9%	0%
	$II_{v}$	59 (±19.7)	34	58.8%	14.7%	26.5%
	$III_{r}$	19.1 (±4.1)	34	97.1%	2.9%	0%
	$IV_{v}$	18.4 (±5.3)	34	94.1%	5.9%	0%
	$IV_{r}$	19.1 (±4.1)	34	97.1%	2.9%	0%
	$V_{r}$	7 (±1.7)	34	100%	0%	0%
	$VI_{v}$	14 (±8.8)	34	94.1%	0%	5.9%
	$VI_{r}$	7 (±2.1)	34	100%	0%	0%

**Table 3:** Angular errors with various methods for Vardy’s image set; testing methods are described in Table 1 (*N* is the number of testing points, μ the average of angular errors and σ the 95% confidence interval).

Method		error μ(±σ) deg.	N	$0\leq \varepsilon_{\theta }<45^{\circ }$	$45\leq \varepsilon_{\theta }<90^{\circ }$	$90\leq \varepsilon_{\theta }<180^{\circ }$
with reference compass	*I*	23.31 (±4.8)	169	97.1%	2.9%	0%
	II	33.28 (±9.8)	169	72.19%	24.26%	3.55%
	III	14.31 (±2.9)	169	100%	0%	0%
	IV	13.8 (±3.1)	169	100%	0%	0%
without reference compass	$I_{v}$	33.30 (±12.1)	169	79.88%	11.83%	8.29%
	$II_{v}$	35.15 (±11.2)	169	69.82%	23.08%	7.1%
	$III_{r}$	14.53 (±2.9)	169	100%	0%	0%
	$IV_{v}$	19.56 (±7.4)	169	96.45%	0%	3.55%
	$IV_{r}$	14.53 (±2.9)	169	100%	0%	0%
